# Deep Anterior Lamellar Keratoplasty: Indications, Surgical Techniques and Complications

**DOI:** 10.4103/0974-9233.61214

**Published:** 2010

**Authors:** Farid Karimian, Sepehr Feizi

**Affiliations:** Department of Ophthalmology, Labbafinejad Medical Center, Director of Cornea and Refractive Surgery Service, Labbafinejad Medical Center, Shahid Beheshti University of Medical Sciences, Tehran, Iran

**Keywords:** Anwar's Big-bubble Technique, Deep Anterior Lamellar Keratoplasty, Lamellar Keratoplasty, Melles'Technique, Penetrating Keratoplasty

## Abstract

The concept of lamellar keratoplasty (LK) is not new. However, it had been abandoned and largely replaced by the time-honored technique of penetrating keratoplasty (PK) because LK is technically demanding, time consuming and gives suboptimal visual outcomes due to interface irregularity arising from manual lamellar dissection. Recent improvements in surgical instruments and introduction of new techniques of maximum depth of corneal dissection as well as inherent advantages such as preservation of globe integrity and elimination of endothelial graft rejection have resulted in a re-introduction of LK as an acceptable alternative to conventional PK. This review article describes the indications, different techniques, clinical outcomes and complications of deep anterior LK.

## INTRODUCTION

Traditionally, penetrating keratoplasty (PK) has been commonly performed as the definitive treatment of a variety of corneal pathologies, such as bullous keratopathy, keratoconus, corneal degenerations and dystrophies. This technique of corneal transplantation is a safe and effective treatment, with numerous studies reporting good visual results after surgery.^1^ However, PK breaches the structural and immunological integrity of the eye, which can result in traumatic wound dehiscence and endothelial graft rejection.^2^

To address such risks, deep anterior lamellar keratoplasty (DALK) is often performed. DALK removes and replaces the pathologic corneal stroma while preserving host healthy endothelium, which eliminates the risk of endothelial graft rejection^3^ and has a reduced effect on the endothelial cell count.^1^,^4^,^5^ In comparison to PK, DALK avoids most complications associated with an open system surgery, such as anterior synechia, expulsive hemorrhage and endophthalmitis.^6^ The criteria for donor cornea tissue selection is less stringent in DALK when compared to PK.^6^

Gasset presented the first series suggesting the use of deep lamellar keratoplasty.^7^ In this series, Gasset described the outcomes of full-thickness grafts, stripped of Descemet's membrane (DM), transplanted into relatively deep lamellar beds in a group of keratoconus patients.^7^ Eighty percent of the patients in Gasset's series achieved best-corrected visual acuity (BCVA) of 20/30 or better. The technique of air-assisted manual dissection DALK, considered the predecessor of other techniques of deep LK, was first introduced by Archilla in 1984.^8^ Over time, a variety of surgical approaches have been proposed for DALK.

The purpose of this article is to review indications, various surgical techniques, clinical outcomes and complications after DALK. A review of the literature was performed by entering the keywords “deep anterior lamellar keratoplasty” and “DALK” in PubMed and reviewing all articles written in English containing these keywords.

## INDICATIONS

The most common indication for DALK is likely keratoconus because the patients benefit the most from preserving their own endothelium. The good outcomes in keratoconic patients has led corneal surgeons to apply this technique of two other pathologies sparing the corneal endothelium. Therefore, the indications for DALK have expanded to other corneal ectasia (pellucid marginal degeneration and post-LASIK ectasia), stromal dystrophies (lattice, macular and granular), stromal opacities and scar and active corneal ulcer and perforations. Generally, DALK can be considered for all corneal pathologies other than those pathologies affecting the endothelium (aphakic and pseudophakic bullous keratopathy, Fuchs’ endothelial dystrophy, iridocorneal endothelial syndrome and posterior polymorphous dystrophy).

PK has been suggested for the elderly patients with corneal pathologies due to the similarity of the graft life-span and patient life expectancy.^9^ However, the elderly are more prone to trauma; DALK, if possible, is still preferable in these patients.

### Keratoconus

The effectiveness of DALK for keratoconus patients has been extensively studied as it is the most common indication for corneal transplantation in some countries.^10^–^13^ These patients, ranging from 20 to 40 years of age need a reliable and effective method of corneal transplantation lasting their lifetime. Acceptable visual acuity of 20/40 or better has been reported in 77.8-92.3% of keratoconus patients after DALK.^14^–^16^ Visual outcomes, contrast sensitivity and higher-order aberrations after DALK are comparable to those after PK in keratoconus patients.^17^–^19^ In contrast to this observation, some studies have reported that DALK yields visual outcomes inferior to PK.^1^,^5^,^20^ The thickness and (smooth) texture of the residual stroma play an important role in this regard. A residual stromal thickness above 20 µm may cause visual acuity to deteriorate.^21^

### Pellucid marginal degeneration

In patients with PMD who are not correctable with spectacles, are contact lens intolerant or when the ectasia is severe enough to preclude contact lenses, surgical intervention such as wedge resection and corneal transplantation can be considered. However, using PK in such cases can result in graft failure in 20% of the eyes due to the proximity of the donor tissue to the limbus, including a peripheral thin area with a high risk of immunologic rejection resulting in endothelial cell loss.^1^ DALK combines the advantages of PK with those of LK while avoiding the disadvantages of both.^22^

### Progressive Post-LASIK keratectasia

Currently, PK has been the preferred approach for progressive post-laser *in situ* keratomileusis (LASIK) keratectasia (PPLK) with successful results.^23^,^24^ The promising outcomes of DALK in the management of keratoconus have led some surgeons to believe that DALK would be a useful approach for iatrogenic keratectasia.^25^–^27^ The technique of DALK is very similar to that previously reported for keratoconus. For post-LASIK cases, the LASIK flap is removed following recipient trephination as the presence of flap makes estimation of the depth of the uncut corneal stroma difficult when injecting air, and the air invades the interface due to the lower resistance. The big-bubble can be successfully formed in post LASIK cases with a final clear graft and significant improvement in best spectacle corrected visual acuity (BSCVA).^27^

### Hereditary stromal dystrophies

Recently, DALK has been performed for cases of corneal stromal dystrophies with normal endothelium.^28^–^32^ Patients with avellino, lattice and granular corneal dystrophies are good candidates for DALK because this technique maximizes depth of dissection, removing diseased stroma as far as DM and providing patients with acceptable visual acuity.^28^,^29^ However, DALK is not suitable for macular corneal dystrophy due to the involvement of the deeper layers of the stroma and possibly the endothelium. The stromal/ endothelial involvement would lead to a higher rate of endothelial cell attrition and fragility of DM.^29^ This observation is consistent with reports of higher recurrence of macular dystrophy in donor tissues that have undergone DALK^30^,^31^ compared to PK.^33^ The higher recurrence rates may be attributed to abnormal keratocytes that remain in the recipient bed after DALK.^31^–^33^

### Corneal stromal scar

The indications of DALK have extended to corneal stromal scars sparing DM (e.g. post-infectious opacity). Anwar's big-bubble technique was applied to corneal stromal opacities following herpetic keratitis. Bared DM and BSCVA ≥20/30 were achieved in 86.5 and 80% of the cases, respectively.^34^ In the previous study, acyclovir was used prophylactically and no participants of the study experienced recurrent episodes during the follow-up period.^34^,^35^

DALK can also be used for corneal stromal scars due to previous surgeries. A successful result has been reported after DALK was performed for an extensive recurrent pterygium invading more than two-thirds of the cornea.^36^

### Infectious keratitis

DALK appears to be superior to PK in infectious corneal ulcers if it is possible to completely remove the infected stroma and reach a clear bed through deep dissection. Advantages of DALK in infectious keratitis include a lower risk of intraocular extension of infectious organisms at the time of surgery and the potential for improved graft survival rates due to the elimination of endothelial rejection reaction. Endothelial rejection reaction is very common following PK in the presence of inflammation and vascularization. Anshu *et al*.^37^ performed three types of keratoplasty, including modified Anwar's big-bubble DALK, manual dissection DALK and conventional PK for advanced bacterial, fungal or acanthamoeba keratitis, which remained unresponsive to medical therapy with risk of extension to the limbus or anterior chamber. DALK and PK eradicated infection in 84.6 and 88% of the cases, respectively. Recurrence of primary infection occurred in 15.3% of the DALK and 12% of the PK group. The recurrence occurring after DALK was observed in the manual-dissection subgroup, while none of the eyes undergoing Anwar's big-bubble technique experienced a recurrent infection. This observation may indicate complete stromal removal by big-bubble DALK, completely eradicating the infection. BSCVA was significantly better after DALK and graft survival rates were 90 and 78.4% after DALK and PK, respectively, in Anshu's study.^37^ Six eyes in the PK group but none in the DALK group developed severe endophthalmitis.^37^ However, the advantages of DALK for infectious keratitis in this study^37^ cannot be attributed solely to techniques of keratoplasty. Surgical intervention in the DALK group was performed earlier and in the less-severe form of corneal ulcers.^37^ Recent reports on performing DALK in cases of non-perforated microsporidial,^38^ acanthamoebal^39^ post-LASIK mycobacterial^40^ and gonococcal^41^ keratitis have shown successful outcomes.

### Tectonic indication

Performing PK in an emergency situation usually has an ominous prognosis and is more likely to fail. DALK can be used to restore the globe integrity when there is an area of thinning or a small perforated ulcer. In the case of descematocele, DM has already been exposed and dissection can be started at this site using a blunt spatula or by viscodissection. However, paracentesis should be performed to soften the anterior chamber and reduce the risk of perforating the DM.

DALK, using manual dissection with the aid of intrastromal air or fluid injection, has been used for a small and peripherally located corneal perforation.^42^,^43^

## CONTRAINDICATIONS

Endothelial dysfunction is an absolute contraindication for DALK. Deep scars involving DM over the entrance pupil and pre-existing defects and breaks in the DM (e.g. acute hydrops) are relative contraindications. It is still possible to perform pre-descemetic DALK in the latter conditions as moderate reduction of vision caused by focal scarring of the DM may be an acceptable compromise to full replacement of largely healthy endothelium. Furthermore, defects in DM can be avoided by leaving a thin layer of stroma in place.

## SURGICAL TECHNIQUES

LK has evolved from conventional intrastromal dissection leaving a large amount of corneal stroma in place to DALK, in which corneal stroma is removed down to DM. In an attempt to simplify identification of the DM or pre-Descemet's deep stromal plane, perform a complete and safe dissection of the overlying corneal stroma and obtain a smooth host surface of uniform thickness. Several techniques for DALK have been proposed. Each technique has its own advantages and disadvantages. Following are brief descriptions of different techniques of dissection that are commonly used by corneal surgeons.

### Layer-by-layer manual dissection

Although recent techniques of DALK have been developed to remove the corneal stroma with ease and success, the basic technique of layer-by-layer manual dissection is still useful in some cases such as pre-existing corneal perforation, strong stroma to DM adhesion (e.g. deep stromal scarring) or inadequate visualization. A partial trephination of approximately 2/3 of the total corneal thickness is performed, followed by stromal removal using a bevel-up crescent knife. Layer-by-layer stromal dissection and resection is repeated as one approaches DM. In spite of being effective, this procedure is technically challenging, time consuming, leaves a relatively rough surface and has a high rate of perforation of DM.^44^

### Air-assisted manual dissection (Archila technique)

Introduced by Archila in 1984,^8^ this technique is considered the predecessor of other techniques of maximum depth dissection, such as Anwar's big-bubble technique. After partial thickness trephination, intrastromal air is injected until the cornea becomes opaque and then manual deep dissection is carried out down to the DM, which appears clear using either a sharp crescent or a blunt spatula. This step can be repeated as long as microbubbles are visible, making sure that there is still a layer of stroma that protects the DM against perforation.

### Air-guided deep stromal dissection (Melles’ technique)

Melles *et al*.^45^ introduced a technique of dissection in which the anterior chamber aqueous is completely replaced with an air bubble to generate a mirror reflection of the spatula inserted into the stromal pocket. The difference in the refractive index between air and corneal tissue creates a mirror image aiding in determining the depth of dissection and locating DM. In this technique, a half-depth scleral incision, 5.0 mm in width, is made 1-2 mm from the limbus at the 12-o'clock surgical position using a guarded diamond knife set at 350 microns. A sclerocorneal tunnel is dissected extending 1.0 mm into the clear cornea. Through the scleral pocket, a beveled spatula is inserted and gradually advanced into the deep stroma until the mirror reflex of the tip of the spatula narrows to a fine line indicating a corneal depth of about 95%. The blunt spatula is advanced over 360 beyond the border of the ongoing trephination, usually an area that covers 9 mm diameter. Subsequently, the air bubble is partially evacuated and the DM is kept away from the overlying stroma using an ophthalmic viscoelastic device (OVD) to displace the posterior corneal layers toward the iris to avoid damaging these layers during trephination. The preparation of the recipient bed is completed by trephination and excision of the anterior stroma.

### Anwar's big-bubble technique

**Figure 1:**
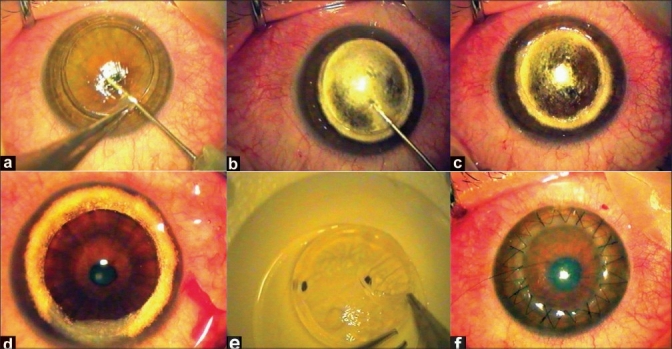
(a) Air injection deep into the stroma with a bevel-down 27-G needle; (b) Round big-bubble formation passing the trephination borders; (c) Formed big-bubble; (d) Exposed Descemet's membrane after removal of the corneal stroma; (e) Removal of donor Descemet's membrane; (f) Conclusion of deep anterior lamellar keratoplasty with the combined suturing technique

The big-bubble technique introduced by Anwar and Teichmann^46^ provides a planned, safe, quick and consistent exposure of DM by the injection of air deep into the stroma. The surface of the DM appears smooth after successful stromal resection. Approximately 80% of corneal thickness is trephined and a 27- or 30-guage needle (bevel facing downward), attached to a 5-cc syringe is inserted into the deep stroma aiming toward the center of the cornea [Figure 1a and b]. Air is gently injected into the deep stroma until a round, well-demarcated big-bubble is formed extending to the borders of trephination [Figure 1c]. After big-bubble formation, debulking of the anterior two-thirds of the corneal stroma is performed with a crescent blade. This is followed by a peripheral paracentesis and excision of the remaining stroma using blunt scissors [Figure 1d].

### Viscoelastic dissection technique

In this technique, the cornea is partially trephined to approximately two-thirds of its thickness. This step is followed by anterior stromal resection and air is injected into the anterior chamber. Using a semi-blunt spatula, the remaining stroma is dissected down to the DM and a small pocket is created. OVD is then injected into the pocket to complete the detachment of DM from the posterior stroma. The procedure of recipient preparation is completed by excising the overlying stroma.^47^

### Hydrodelamination

Sugita and Kondo^19^ used hydrodelamination in which saline solution was injected into the cornea to enhance identification and removal of the deep stromal fibers. However, achieving an actual cleavage plane over DM is difficult. A modification of this technique has been introduced.^48^ After partial trephination of the recipient cornea, balanced salt solution is injected with a 30-gauge needle in the four quadrants to make the trephined area completely opaque. A limbal paracentesis is performed to lower the intraocular pressure and to decrease the incidence of perforation during dissection. Then, lamellar dissection is performed down to the DM.

The aforementioned procedures have some steps in common, making it possible for cornea surgeons to master two or more techniques for maximal depth of dissection. Familiarity with different techniques helps surgeons choose an appropriate plan for different situations. For example, in the case of deep stromal scar or breaks in DM (previous hydrops), Melles’ technique is preferred as any attempt to bare the DM (e.g. using Anwar's big-bubble technique) in such eyes, resulting in DM rupture. Additionally, it may be necessary to change from one technique to another due to intraoperative complications. For example, the success rate of the big-bubble technique can be enhanced by injecting OVD or fluid into the residual posterior stroma after unsuccessful air injection.

### Donor preparation

The recipient trephine size is selected to be as large as possible. A 7.5-mm trephine is selected for a corneal diameter ≤10 mm and an 8.0-mm or larger trephine is chosen for corneal diameters >10 mm. The disparity between recipient and donor trephine size is based on the vitreous length. A disparity of 0.25 mm is selected for a vitreous length of ≥16.0 mm and, to reduce post-operative hyperopia, a donor size 0.50 mm larger than the recipient bed is appropriate if the vitreous length is <16.0 mm (Unpublished observation). The same-size or undersized donor tissue is not advisable as it can lead to problems with flat graft surface, including inadequate tear film distribution, and hence delayed epithelialization, post-keratoplasty hyperopia, difficult contact lens fitting and severe interface wrinkling.^16^,^49^ The retention of removal of donor DM during preparation of donor tissues remains controversial. If the donor DM is left in place it may delay wound healing at the recipient-donor interface.^50^ Additionally, in cases of recipient DM perforation a non-resorbing pseudo-anterior chamber is more likely.^50^ Additionally, the donor endothelium may present potential antigens for immunological rejection. We personally prefer to remove the DM during donor tissue preparation [Figure 1e].

If an OVD is used over DM during recipient preparation, thorough irrigation and removal is strongly advocated before proceeding to donor suturing to prevent potential pseudoanterior chamber formation.

### Suturing techniques

Both merseline and nylon 10/0 sutures can be used. Different suturing techniques have been applied to secure a donor cornea to the recipient bed. However, separate sutures alone or in combination with a single running suture seem to be more appropriate [Figure 1f]. It is important that sutures encompass at least 90% of both donor and recipient thickness to prevent post-operative complications such as early suture loosening or cheese-wiring.

## CLINICAL OUTCOMES

The techniques of dissection as well as surgeon's experience are main factors in determining the rate of true DM exposure. Performing four different techniques of maximum depth dissection on keratoconic eyes, Sarnicola *et al*.^51^ found the highest rate (60%) with Anwar's big-bubble technique. Supplemented with viscoelastic dissection at the same session in the case of unsuccessful air injection, this rate increased to as high as 77%.^51^ In the Sarnicola study,^51^ viscodissection technique was the second most successful technique in baring the DM, with a rate of 58%, followed by the Tsubota technique (10.5%) and hydrodelamination (7%).

### Visual acuity

Post-operative BCVA of 20/40 or better has been reported in 77.8-92.3% of the eyes having undergone DALK with the big-bubble technique.^14^–^16^ Compared to PK, there are controversial reports on the superiority of DALK in terms of post-operative visual acuity. Panda *et al*.^5^ obtained better visual acuity after DALK, while the results reported by Tsubota *et al*.^44^ favored PK. Watson *et al*.^1^ and Shimazaki *et al*.^18^ did not find any significant difference between the two techniques in terms of BCVA and contrast visual acuity. Similarly, Javadi *et al*.^52^ showed that there was no significant difference between the Anwar big-bubble technique and PK in keratoconus patients in terms of post-operative BCVA, contrast sensitivity and higher-order aberrations. These conflicting results can be attributed to the irregularity at the host-donor interface and the thickness of the residual stroma.^53^ Comparing the eyes with keratoconus that underwent DALK using Melles’ technique with PK, Ardjomna *et al*.^21^ found that the visual acuity of the DALK group was similar to that of the PK group only when the recipient corneal bed thickness was <20µm.

### Refractive outcomes

Similar to PK, post-operative myopic and astigmatic refractive errors remain the main cause of patient dissatisfaction after DALK. A wide range of post-operative refractive error from –13.0 D to + 7.0 D has been reported in peer-reviewed literature.^1^,^17^,^48^,^54^ Feizi *et al*.^14^ achieved a mean post-operative spherical equivalent refractive error of –3.41 D (range, –11.75 D to + 3.00 D) after DALK for keratoconus.^14^ In comparison to PK, conflicting results have been reported with some studies^5^,^20^,^55^ reporting lower astigmatism and other studies^1^,^18^ reporting slightly higher astigmatism after DALK. Performing DALK using the big-bubble technique in eyes with keratoconus, Fonata *et al*.^54^ reported topographic astigmatism of >4 D in 16% of the patients. Noble *et al*.^32^ reported that 24.6% of the patients who underwent DALK with the Melles technique for different corneal pathologies developed refractive astigmatism of >5 D post-operatively. Feizi *et al*.^14^ observed astigmatism ≥4 D in 34.4% of the patients undergoing DALK.

### Endothelial cell loss

The loss of endothelial cells occurs immediately after DALK and remains almost constant thereafter. van Dooren *et al*.^56^ observed a rate of endothelial cell loss of 14.3% over a 2-year period after DALK with the Melles’ technique. The majority of cell attrition occurred within the first 6 months post-operatively (11.1%), reaching a physiologic rate of 1-2% per year thereafter. In comparison, a rate of 33% has been reported over the same time period after PK.^18^ Endothelial cell loss after DALK is chiefly attributed to surgical trauma and, in the early post-operative period, it does stabilize.^57^ In contrast, cell loss after PK may result from the surgical trauma, endothelial cell redistribution and episodes of allograft rejection.^57^

### Histopathologic findings

DALK with different techniques of maximum depth dissection is used to completely remove the stroma down to the DM. However, retention of residual posterior stroma appears to be inevitable and the dissection plane is actually located at the most posterior stroma layers instead of between DM and stroma.^47^,^58^,^59^ Performing histopathologic examination on recipient DMs taken after successful big-bubble formation, Jafarinasab *et al*.^60^ observed a thin layer of stroma ranging from 6.4 to 25.8 µm that remained attached to the DM.

Alterations in the density and distribution of keratocytes after DALK have been reported by several studies with confocal microscopy. Using confocal microscopy in seven patients with a history of keratoconus, Balestrazzi *et al*.^61^ reported a slightly reduced density of keratocytes with irregular distribution in the anterior stroma and highly reflective particles and linear hyporeflective microfolds at the interface 18 months after air-guided manual DALK. Marchini *et al*.^62^ reported changes in the deep stromal interface characterized by discontinuity of tissue and cellular stromal architecture, absent or reduced keratocyte density and variable background extracellular reflectivity. However, they did not observe statistically significant changes in anterior and posterior mean keratocyte density or visible scarring at the interface. Farias *et al*.^63^ and Feizi *et al*.^64^ reported a lower density of keratocytes after deep anterior LK using the big-bubble technique in patients with keratoconus. Additionally, there were hyporeflective striae in the rear stroma representing microfolds and sheets of moderate- to high-reflective amorphous deposits at the interface.^64^

## COMPLICATIONS

Some post-operative complications are similar between DALK and PK. For example, post-operative epithelial abnormality and high refractive error are observed after both techniques of transplantation. DALK largely eliminates some complications commonly observed after PK, such as wound leakage and endothelial graft rejection reactions. However, folds within DM, DM perforation, pseudoanterior chamber formation and interface keratitis exclusively develop after DALK.

### DM perforation

Several published studies^16^,^17^,^19^,^44^,^65^ have reported DM perforation during DALK, ranging from 4 to 39.2%. This rate appears to depend on surgeon experience, indications for keratoplasty and surgical techniques. Keratoconus patients are more prone to DM ruptures than patients with other corneal disease.^66^ Microperforation rates seem to be highest with manual layer-by-layer dissection (26.3%) and lowest with the Anwar big-bubble technique (5.48%). Microperforation rates with hydrodelamination (7.3%) and viscodissection (8.3%) techniques are low.^51^

Perforation of DM can occur during any step of surgery, including trephination, stromal excision and donor suturing. The moment of perforation is crucial for the completion and the success of DALK. Early perforations lead to a greater residual stroma and hence slower visual rehabilitation due to haziness of the stromal interface. Also, the size of perforation is a determining factor for endothelial cell loss; a large hole within the DM results in a flat anterior chamber requiring multiple air injections, which is associated with a greater severity of endothelial damage.^67^ Management of DM perforation depends on the size and location of the defect and the step of surgery at which the perforation occurs. If perforation occurs initially during trephination, it is possible to tightly suture the site of perforation and continue lamellar dissection. Notably, in this area, sutures that fix donor tissue to the recipient bed should be full-thickness, including recipient DM. If perforation occurs during stromal excision, the site of perforation should be dissected at the end, and it is preferable to leave a thin layer of posterior stroma over this area to seal the perforation. At the end of surgery, the anterior chamber is partially filled with an air bubble. For perforations that occur during suturing, injection of air into the anterior chamber at the conclusion of surgery is usually sufficient.

### Pseudoanterior chamber

**Figure 2:**
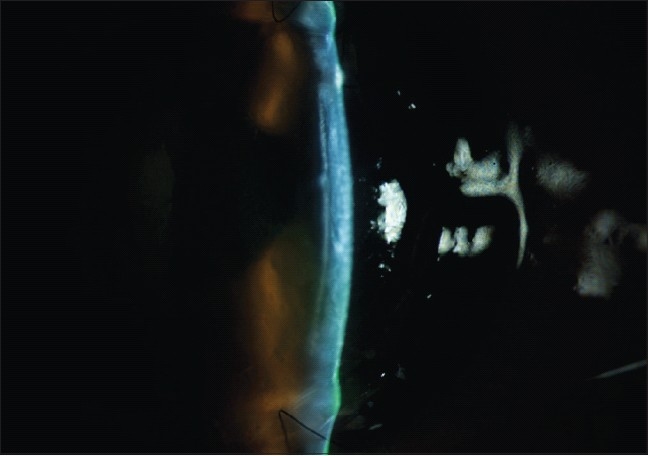
Pseudo-anterior chamber formation due to microperforation of the Descemet's membrane

Pseudoanterior chamber usually occurs secondary to breaks in the DM [Figure 2]. Occasionally, retention of an OVD can lead to a detached DM.^68^ A shallow pseudochamber may be self-limited and may resolve after a few days. However, large pseudochambers may persist for weeks and may require surgical intervention. Surgical correction of pseudoanterior chambers may be performed by injection of air or expandable gases such as sulfur hexaflouride (SF6). Possible complications of this intervention include pupillary block, iris atrophy, fixed dilated pupils and cataract formation. To prevent these complications, the pupil must be dilated with potency-weak mydriatic prior to injection, followed by anti-glaucoma medications. If a single injection of air is not enough to correct the problem, the process can be repeated until attachment is achieved.

### Fixed dilated pupil (Urrets-Zavalia syndrome)

**Figure 3:**
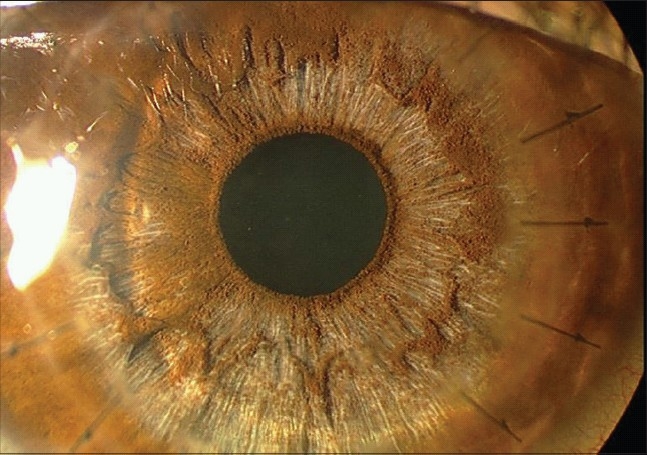
Iris stromal atrophy following managed post-operative pupillary block

A fixed dilated pupil is not a common complication of PK for keratoconus. Although the precise origin of the syndrome is uncertain, it has been proposed that ischemia of the iris can develop after raised intraocular pressure. Intracameral air/gas injection to seal intraoperative DM perforation to treat pseudoanterior chamber may cause pupillary block leading to iris atrophy [Figure 3], iridoplegia, posterior synechia and anterior subcapsular cataract.^69^ If it is necessary to leave an air bubble in the anterior chamber, less than half of the chamber should be filled with air and/or a peripheral iridotomy should be performed to prevent this complication.

### Interface wrinkling

**Figure 4:**
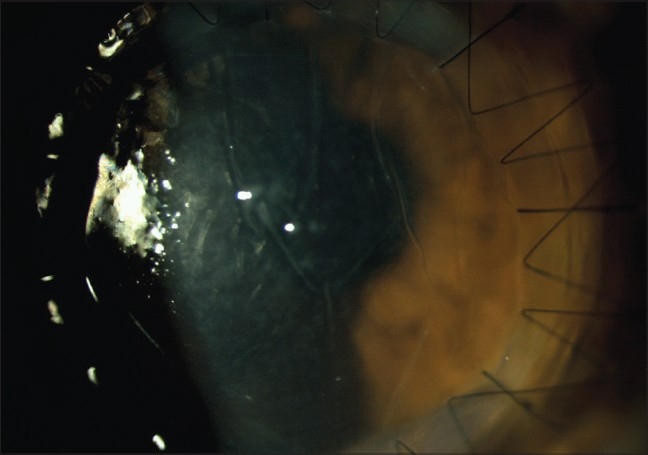
Interface wrinkling following deep anterior lamellar keratoplasty

Folds in the DM following DALK are usually transient and improve over time. The folds are often located peripherally and have no impact on vision [Figure 4]. Central folds can decrease the visual acuity^70^ likely due to an increased level of higher-order aberrations.^71^ A mismatch between the donor button and the recipient bed size is responsible for folds in the DM; hence, oversizing the donor button by 0.25-0.50 mm is recommended to prevent this complication.

### Graft rejection reaction

Although DALK eliminates the risk of endothelial rejection, other types of graft rejection (subepithelial and stromal) may still develop, with an incidence of between 3 and 14.3%.^1^,^2^,^72^ The clinical course of subepithelial and stromal graft rejection after DALK are very similar to that of PK. Frequent topical steroid usually leads to reversal of the rejection reactions.^73^ Although rejection after LK is easy to control, subepithelial and stromal graft rejections must be treated appropriately to prevent less-severe yet important complications such as suture abscess and graft vascularization that can lead to poor visual outcomes and even lamellar graft failure.^74^

### Interface vascularization and opacification

**Figure 5:**
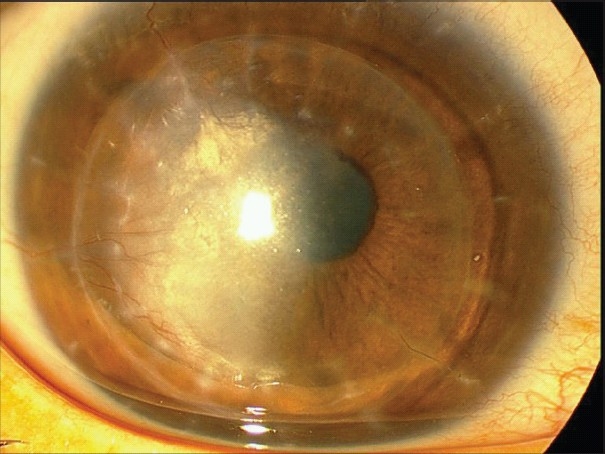
Vascularization of the interface with protein extravasations and opacification

The occurrence of surface and suture complications may stimulate vascularization of the graft and interface. This vascularization leads to early suture loosening, appositional problems and the need for resuturing. The primary indication for DALK has an influence on this complication. Extensive vascularization may result in lipid and protein extravasations leading to interface opacification [Figure 5] and hence visual acuity reduction.

### Suture-related complications

**Figure 6:**
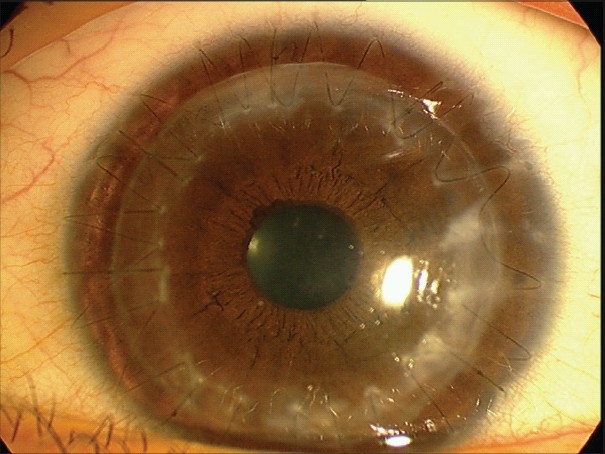
Suture-induced reaction, cheese-wiring and loose sutures after deep anterior lamellar keratoplasty

Suture-related complications, such as sterile reactions, early suture loosening, cheese-wiring and vascularization, [Figure 6] as well as overhanging donor-recipient junction appear to be more common after DALK in comparison to PK (unpublished observation). These complications can be reduced or even prevented by appropriate suture depth (90% of thickness on both recipient and donor sides), length and tension.

### Interface keratitis

The interface left during DALK is a potential dead space and introduction of microorganisms intraoperatively can proliferate within this space without a host immune response. The most common microorganism retrieved from DALK-interface keratitis is Candida.^75^ Candida infection occurs due to donor corneal contamination or by the indigenous microflora of the conjunctiva and ocular adnexa. After transplanting a contaminated donor cornea to the recipient bed, development of endophthalmitis is avoided or delayed by the presence of DM separating the site of infection from the intraocular structures. However, the location of infection may make it difficult to obtain specimens for culture and prevent adequate penetration of topical, intraocular and systemic antibiotics, making conservative treatment more likely to fail. The first two cases of Candida keratitis after DALK required PK.^75^

## CONCLUSION

DALK appears to be an acceptable alternative to PK in stromal corneal diseases because it retains the advantages of both lamellar and full-thickness corneal transplantation and eliminates the drawbacks of the interface created during conventional lamellar keratoplasty. However, there are still some aspects of DALK that require further study. More extensive studies with longer follow-up periods are required to understand the advantages and disadvantages of DALK.
